# Taste Sensitivity Is Inversely Associated with Body Mass Index (BMI) Independently of Caloric Intake: Evidence from Sensory and Genetic Analyses

**DOI:** 10.3390/nu18152459

**Published:** 2026-07-27

**Authors:** Melania Melis, Silvia Deligia, Lala Chaimae Naciri, Iole Tomassini Barbarossa

**Affiliations:** Department of Biomedical Sciences, University of Cagliari, 09042 Monserrato, Italy; silvia.deligia@unica.it (S.D.); l.naciri@studenti.unica.it (L.C.N.); tomassin@unica.it (I.T.B.)

**Keywords:** taste sensitivity, body mass index, *TAS1R* genes, *CD36*, *GNAT3*, nutrigenetics, eating behavior, energy intake

## Abstract

**Background/Objectives**: Taste perception has emerged as a key determinant of eating behavior and metabolic regulation, but its relationship with body mass index (BMI) remains incompletely understood. We investigated the relationships among global, sweet, and lipid taste sensitivity; sweet- and lipid-taste-related polymorphisms; caloric intake; and BMI. **Methods**: Taste sensitivity was assessed using taste strips (overall and sweet) and detection thresholds for fatty acids (oleic, linoleic, and palmitic acids). Genotyping of polymorphism genes was conducted. Pearson correlation analyses examined bivariate associations between taste variables and BMI. Multiple regression models were performed to identify independent predictors of BMI and to evaluate the mediating role of caloric intake. **Results**: A strong inverse correlation was found between total and sweet taste sensitivity and BMI, particularly in super-tasters (STs) and participants with the sensitive genotype of sweet-taste-related polymorphisms. Similarly, greater sensitivity to fatty acids was associated with lower BMI, specifically in non-tasters (NTs) and participants with the insensitive genotype of *CD36* polymorphisms. In multiple regression models, overall and lipid sensitivity were the most significant predictors of BMI, which were inversely associated with it. *TAS1R2* and *TAS1R3* also showed independent effects. Importantly, caloric intake was not retained in the final model. **Conclusions**: Taste sensitivity is inversely associated with BMI independently of caloric intake. These findings suggest that the gustatory system could influence body weight through mechanisms beyond energy consumption, probably involving food choice and extra-oral receptor-mediated metabolic regulation. Taste perception and related genetic factors may represent important targets for personalized nutrition and obesity prevention strategies.

## 1. Introduction

Obesity represents one of the most pressing global health challenges, characterized by a complex interplay of genetic, environmental, and behavioral factors. Traditionally, body mass index (BMI) has been primarily linked to energy imbalance, with excessive caloric intake considered a key driver of weight gain. However, increasing evidence suggests that individual variability in eating behavior and metabolic outcomes cannot be explained solely by differences in caloric intake, indicating the involvement of additional regulatory mechanisms [1,2].

Among these, taste perception has emerged as a critical determinant of food choices, dietary behavior, and, ultimately, body weight regulation [3,4,5]. Taste serves as the primary interface between the individual and food, providing rapid information about nutrient composition and energy density and guiding both acceptance and intake [6]. Variability in taste perception has been shown to influence dietary preferences, including the consumption of sugar- and fat-rich foods, which are strongly associated with obesity risk [7]. However, other studies did not support these relationships [8,9].

Sweet taste perception plays a central role in the evaluation of carbohydrate-rich, energy-dense foods. However, the view that a “sweet tooth” leads to obesity through excessive sugar consumption is overly simplistic [10]. Most foods perceived as “sweets” are actually energy-dense, high-fat foods [11] and epidemiological studies have shown that the consumption of purely sweet foods is not associated with obesity [12,13]. The perception of sweetness is mediated by the heterodimeric receptor composed of T1R2 and T1R3 subunits, encoded by *TAS1R* genes, together with downstream signaling components such as α-gustducin, encoded by the *GNAT3* gene. Genetic polymorphisms in these receptors have been linked to differences in sweet taste sensitivity, sugar intake, and BMI, suggesting that genetic variation in taste receptors contributes to interindividual differences in energy balance [14,15]. In particular, the single-nucleotide polymorphism (SNP) *rs35874116* (C/T) of the *TAS1R2* gene has been associated with sugar intake [16,17,18] and with sweet taste sensitivity [19], with the CC genotype showing higher sensitivity [19]. However, other studies did not show this association [15,20]. The polymorphism, *rs12033832* (G/A) of *TAS1R2* has been shown to affect sweet taste perception and sugar intake in a BMI-dependent manner [15]. Individuals with a BMI ≥ 25 and two G alleles reported lower intensity ratings and higher sugar consumption than those homozygous for the A allele [15]. The *rs307355* SNP in the *TAS1R3* gene’s promoter region has been associated with sweet taste sensitivity, with the C allele linked to higher receptor expression and greater sweet sensitivity [20]. In addition, *GNAT3 rs7792845* has been linked to sweet taste perception, with CC carriers showing higher sweet sensitivity than CT or TT genotypes [21]. Moreover, sweet taste receptors, as well as α-gustducin, are expressed not only in the oral cavity but also in extra-oral tissues, such as the gastrointestinal tract and pancreas, where they participate in glucose sensing, hormone secretion, and metabolic regulation [22,23,24].

Similarly, fat perception has been increasingly recognized as a distinct sensory modality, often referred to as “fat taste” or oleogustus [25,26]. Long-chain fatty acids can be detected in the oral cavity through specific receptors, including CD36, which plays a major role in lipid sensing [27,28,29]. The *CD36 rs1761667* SNP affects CD36 protein expression, with the AA genotype associated with lower expression levels compared with G-allele carriers [30,31]. This CD36 SNP has been associated with fat taste perception and preference [32,33,34,35,36]. Differently, the *rs1984112* SNP in the *CD36* gene has been mainly associated with lipid metabolism, with the AA genotype linked to higher fasting triglyceride levels and an enhanced postprandial lipid response after a high-fat meal [37]. Variations in fat taste sensitivity have been associated with differences in fat intake and obesity risk. Individuals with reduced sensitivity to dietary fats tend to exhibit a higher preference for high-fat foods and an increased BMI, suggesting that fat taste perception may influence body weight regulation [34,38].

In addition to modality-specific effects, overall taste sensitivity has also been proposed as an important determinant of eating behavior [39,40]. Individuals with heightened global taste perception may experience stronger sensory responses to food stimuli, potentially leading to lower intake and improved energy regulation [6]. However, the relationship between global taste sensitivity and BMI remains incompletely understood, and findings are often inconsistent across studies [6,7]. In addition, the genetic ability to taste the oral marker 6-*n*-propylthiouracil (PROP), which has been used as an indicator of general taste sensitivity in numerous studies [35,41,42,43,44,45,46,47], has also been associated with changes in general taste sensitivity, BMI (with an opposite trend in subjects with obesity compared to subjects with normal weight), plasma endocannabinoids and lipid parameters, which could be interrelated to modifications in dietary patterns and body fat distribution [3,48].

Therefore, this additional layer of complexity provided by the role of variants in sweet and fatty acid taste-related genes, including *TAS1R* and *CD36*, influences both sensory perception and dietary behavior. These genetic factors may contribute not only to differences in taste perception but also to variability in metabolic responses and obesity susceptibility [14,29]. Emerging evidence suggests that taste receptors expressed in extra-oral tissues may play a role in metabolic regulation, supporting the hypothesis that taste perception may influence BMI beyond behavioral dietary mechanisms [24]. Therefore, while taste perception is known to affect food intake, it remains unclear whether its association with BMI is primarily mediated through total caloric intake, or whether taste exerts direct effects on body weight independent of energy consumption.

Based on this evidence, the present study aimed to investigate the relationship between taste sensitivity (global, sweet, and lipid), polymorphisms in sweet- and lipid-taste-related genes, caloric intake, and BMI. Specifically, we sought to determine whether taste sensitivity is directly associated with BMI, whether genetic variation in taste receptors contributes independently to BMI variability, and whether the relationship between taste perception and BMI is mediated by caloric intake.

## 2. Materials and Methods

### 2.1. Subjects

Eighty-six Caucasian participants (27 males, 59 females, age 26.74 ± 8.04) were recruited through public advertisements at the University of Cagliari. All participants were originally from Sardinia, Italy. The exclusion criteria included major diseases (e.g., hypertension, diabetes, and kidney disease), pregnancy or lactation, food allergies, eating disorders, prescribed diet, a history of persistent post-COVID-19 hyposmia and/or ageusia and drugs interfering with taste or smell. The participants’ body mass index (BMI) ranged between 17.5 and 32 kg/m^2^. The BMI of five participants ranged from 17.5 to 18.3 kg/m^2^, indicating mild underweight status. None reported body weight fluctuations exceeding 5 kg during the previous three months, indicating stable body weight at the time of assessment. Participants were instructed to complete a food diary for 1 week. The data were analyzed using WinFood software 4.0, (Medimatica S.u.r.l., Colonnella, TE, Italy), which incorporates a comprehensive food composition database to estimate daily energy intake (kcal/day), including carbohydrate and fat intake, and nutrient composition.

Each participant was informed about the study aims and procedures. They provided a signed consent form. The study was conducted in accordance with the latest version of the Declaration of Helsinki, and the experimental protocol was approved by the University Hospital Company (AOU) Ethical Committee in Cagliari, Italy.

### 2.2. Experimental Procedure

All participants were instructed to abstain, for at least two hours before testing, from consuming any food or beverages (except water), using oral hygiene products, or chewing gum. They had to arrive in the test laboratory 15 min before testing (9.00 AM) to adapt to controlled environmental conditions (23–24 °C; 40–50% relative humidity). In women, the taste tests were always carried out on the sixth day of the menstrual cycle to avoid taste sensitivity changes due to the estrogen phase as shown by Glanville et al. [49]. Each participant was tested in two sessions on consecutive days. During the first session, participants were classified for their taster status and tested for their overall taste sensitivity. Weight and height were measured to calculate the BMI (kg/m^2^), and a sample of whole unstimulated saliva (2 mL) was collected and stored at −80 °C until molecular analyses were completed, as described below. During the second session, fatty acid thresholds (oleic, linoleic, and palmitic acids) were assessed. Stimuli were presented at room temperature, and participants washed their mouths with spring water after each stimulation.

### 2.3. Taster Status Classification

Participants were characterized by their genetic ability to taste the bitterness of the oral marker PROP by using the impregnated paper screening test [50], a procedure widely validated in numerous studies to classify subjects as super tasters (STs), medium tasters (MTs) or non-tasters (NTs) [51,52]. In brief, two paper disks, one impregnated with PROP solution (50 mmol/L) and one with sodium chloride, NaCl (1.0 mol/L), were applied on the tip of the tongue for 30 s. Perceived taste intensity was rated using the Labeled Magnitude Scale (LMS) [53]. Participants who rated the PROP disk below 15 mm on the LMS were classified as NTs, whereas those providing ratings above 67 mm were classified as STs, and all others were categorized as MTs [50]. NaCl ratings were used as a reference in borderline cases [54]. The interstimulus interval was set at 60 s.

Based on their taster group assignments, 35 participants were classified as STs (40.7%), 32 as MTs (37.2%), and 19 as NTs (22.1%).

### 2.4. Taste Sensitivity Measurements

Taste sensitivity to the four primary qualities (sweet, sour, salty, bitter) was assessed using the Taste Strip Test (TST, Burghart Company, Wedel, Germany) [55]. Briefly, 16 filter paper strips impregnated with four concentrations of stimuli that represent four basic taste qualities: sweet (0.05, 0.1, 0.2, and 0.4 g/mL of sucrose); sour (0.05, 0.09, 0.165, and 0.3 g/mL of citric acid); salty (0.016, 0.04, 0.1, and 0.25 g/mL of NaCl); and bitter (0.0004, 0.0009, 0.0024, and 0.006 g/mL of quinine hydrochloride) were presented in a pseudorandomized order (with increasing concentrations within each taste quality). Participants placed each strip on the tongue and identified the perceived taste quality using a list of five descriptors (sweet, sour, salty, bitter, or no taste). Each correct answer was rated 1, and the maximum score for the total taste strip was 16. The interstimulus interval was set at 60 s. We were unable to perform taste sensitivity measurements due to the unavailability of 13 participants.

### 2.5. Fatty Acid Threshold Assessments

The thresholds for oleic, linoleic, and palmitic acid were determined in each participant, without nose clips, using a variation of the staircase method implemented in a 3-Alternative Forced Choice procedure as described by Melis et al. [35]. For each fatty acid, participants received three paper filter disks: two containing 10 µL of mineral oil (control) and one containing the fatty acid amount to be identified. Stimuli were presented in ascending concentrations until the participant correctly identified the target sample in two consecutive trials. Concentrations presented were increased after a single incorrect response and decreased after two consecutive correct responses. The detection threshold was defined as the lowest concentration at which the target sample was correctly identified. The three fatty acids were presented in a randomized order. The interstimulus interval was approximately 1–2 min. We were unable to determine the thresholds for linoleic acid in 4 participants and for palmitic acid in 1 participant due to their unavailability.

### 2.6. Molecular Analysis

Participants were genotyped for selected polymorphisms in sweet- and lipid-taste-related genes, including *TAS1R2* (*rs35874116* and *rs12033832*), *TAS1R3* (*rs307355*), *GNAT3* (*rs7792845*), and *CD36* (*rs1761667* and *rs1984112*).

DNA was extracted from saliva samples using the standard salting-out procedure, and its quantity and purity were evaluated by spectrophotometry at 260 nm (NanoDrop One/One Spectrophotometer, Thermo Fisher Scientific, Waltham, MA, USA). Genotyping was performed using TaqMan^®^ SNP Genotyping Assay (Applied Biosystems by Life-Technologies Italia, Europe BV, Monza, Italy), according to the manufacturer’s instructions (Applied Biosystems, Life Technologies, Milan, Italy). Reactions were carried out in 96-well plates under fast thermal cycling conditions and included 1× TaqMan^®^ Genotyping Master Mix (code: 4371355; Applied Biosystems by Life-Technologies Italia, Europe BV, Monza, Italy), 1× TaqMan^®^ Genotyping Assays (C_55646_20, C_25985586_10, C_188859166_10, C_11836868_10, C_8314999_10, and C_12093946_10, Applied Biosystems by Life-Technologies Italia, Europe BV, Monza, Italy), 10 ng of DNA, and nuclease-free water. Amplification and fluorescence detection were performed using the StepOne™ Real-Time PCR System (Life-Technologies Italia, Europe BV, Monza, Italy), and genotypes were assigned by allelic discrimination analysis (Sequence Detection Software v2.3). Positive and negative controls, as well as replicating samples, were included in all runs. We were unable to determine the genotype of some participants (1 for *rs12033832*, *rs1761667*, and *rs1984112*; 2 for *rs307355*) due to the poor concentration and/or purity of the extracted DNA.

### 2.7. Statistical Analyses

The relationships between the BMI and total taste strips, sweet taste strips, oleic acid threshold, linoleic acid threshold, palmitic acid threshold, total caloric intake, carbohydrate intake, and fat intake were assessed using Pearson linear correlation analysis. The same analysis was conducted according to the three taster groups, according to genotypes of the *TAS1R2 rs35374116*, *TAS1R2 rs12033832*, *TAS1R3 rs307355*, *GNAT3 rs7792845*, *CD36 rs1761667*, and *CD36 rs1984112* SNPs and segregated by sex.

To identify independent predictors of BMI, stepwise multiple regression analyses were performed, including overall, sweet and lipid taste-sensitivity measures, taster status, and sweet- and lipidic-genetic variants as predictors. In addition, to investigate whether caloric intake mediates the relationship between taste perception and BMI, total caloric intake was included as an independent variable in a second regression model. Specifically, forward stepwise regression was chosen because the study aimed to identify the strongest independent predictors of BMI among multiple sensory and genetic variables. Total caloric intake was subsequently added as a theory-driven candidate variable to evaluate whether the associations between taste-related factors and BMI were explained by energy intake. The relative contribution (β), each step R^2^, and the interpretation of each variable that entered the model are reported in the tables.

Statistical analyses were conducted using STATISTICA for WINDOWS (version 7; StatSoft Inc., Tulsa, OK, USA). *p*-values ≤ 0.05 were considered significant.

## 3. Results

### 3.1. Correlation Analyses

**Figure 1:**
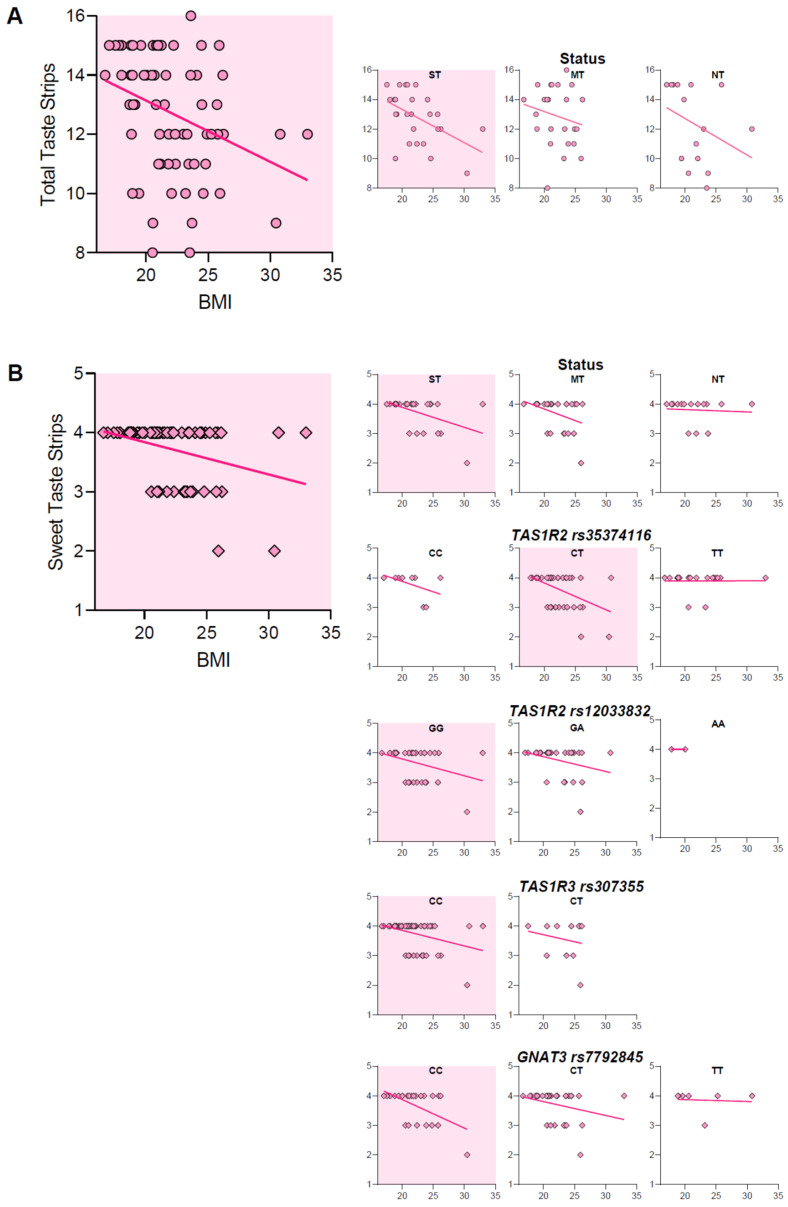
Scatterplots showing the Pearson linear correlation between the total taste strips and BMI in the whole sample and according to the Taster Status of participants (**A**). Scatterplots showing the Pearson linear correlation between the sweet taste strips and BMI in the whole sample and according to Taster Status, *TAS1R2 rs35374116* SNP, *TAS1R2 rs12033832* SNP, *TAS1R3 rs307355* SNP, and *GNAT3 rs7792845* SNP (**B**). Pink area indicates a statistical difference (r > −0.331, *p* < 0.004). Whole sample: *n* = 73; super taster, ST: *n* = 31; medium taster, MT: *n* = 26; non-taster, NT: *n* = 16. Genotypes of *TAS1R2 rs35374116:* CC (*n* = 11), CT (*n* = 39) and TT (*n* = 23); genotypes of *TAS1R2 rs12033832*: GG (*n* = 40), GA (*n* = 30) and AA (*n* = 2); genotypes of *TAS1R3 rs307355* CC (*n* = 62) and CT (*n* = 11) and genotypes of *GNAT3 rs7792845:* CC (*n* = 25), CT (*n* = 38) and TT (*n* = 8).

To explore the bivariate relationships between taste perception and BMI, Pearson correlation analyses were performed. Scatterplots depicting the relationship between total taste strips and BMI in the whole sample and according to the taster status are shown in Figure 1A. A significant inverse correlation was observed between total taste strips and BMI in the whole sample (r = −0.331, *p* = 0.004) and in super tasters (STs) (r = −0.504, *p* = 0.004), while no significant associations were found in medium tasters (MTs) or non-tasters (NTs) (*p* > 0.05). Figure 1B shows the relationship between the sweet taste strips and BMI in the whole sample, across taster groups, and according to *TAS1R2 rs35374116*, *TAS1R2 rs12033832*, *TAS1R3 rs307355*, and *GNAT3 rs7792845* genotype groups. The number of sweet taste strips was inversely correlated with BMI in the whole sample (r = −0.349, *p* = 0.002) and in ST participants (r = −0.457, *p* = 0.009). Significant negative correlations were also found in specific genotype groups, including *TAS1R2 rs35374116* CT (r = −0.479, *p* = 0.002), *TAS1R2 rs12033832* GG (r = −0.365, *p* = 0.021), *TAS1R3 rs307355* CC (r = −0.364, *p* = 0.004), and *GNAT3 rs7792845* CC (r = −0.506, *p* = 0.009). No significant associations were observed in other subgroups (*p* > 0.05).

Pearson correlation analyses were also conducted separately in females and males. Total taste strips and sweet taste strips were inversely associated with BMI in females (A: r = −0.367, *p* = 0.008; C: r = −0.511, *p* = 0.0001) (Figure S1A,C), while no significant associations were observed in males (*p* > 0.05; Figure S1B,D).

**Figure 2:**
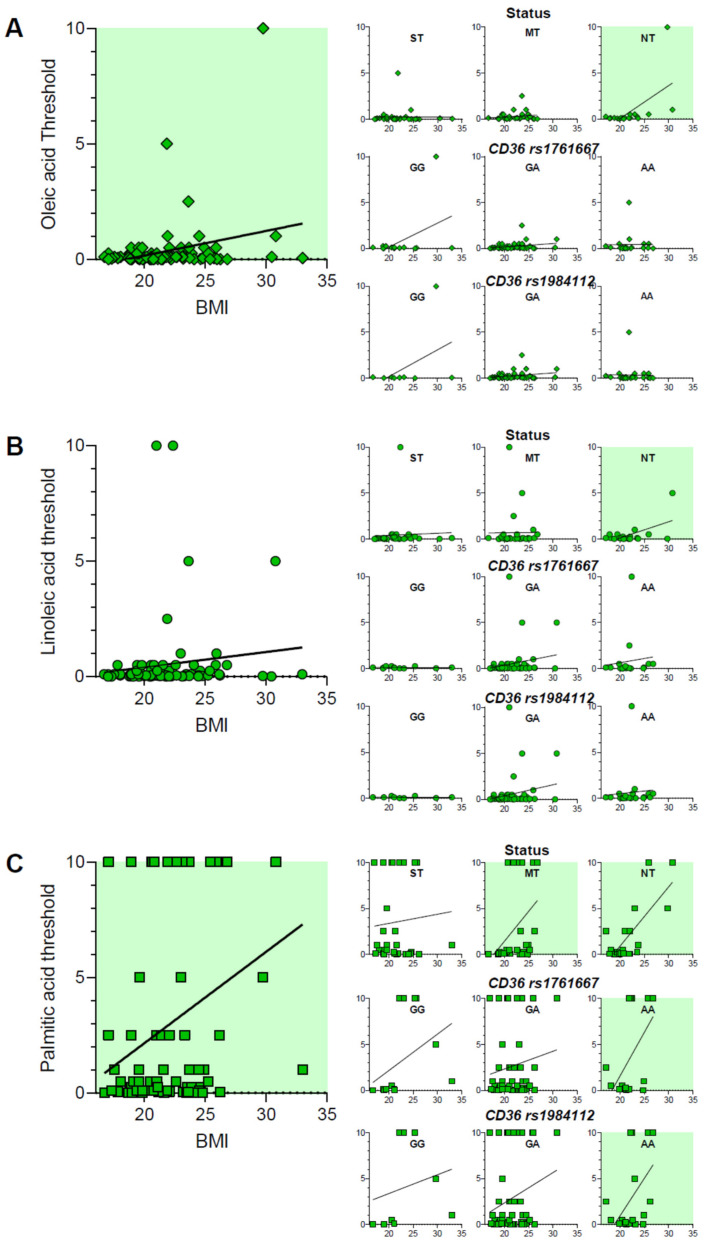
Scatterplots showing the Pearson linear correlation between the BMI and the oleic acid threshold (**A**), linoleic acid threshold (**B**), and palmitic acid threshold (**C**) in the whole sample and in the three taster groups and the *CD36 rs1761667* and *CD36 rs1984112* genotype groups. Green area indicates a statistical difference (r > 0.384, *p* < 0.0165). (**A**): Whole sample: *n* = 86; super taster, ST: *n* = 35; medium taster, MT: *n* = 32; non-taster, NT: *n* = 19. Genotypes of *CD36 rs1761667*: GG (*n* = 14), GA (*n* = 51) and AA (*n* = 20); genotypes of *CD36 rs1984112*: GG (*n* = 9), GA (*n* = 52) and AA (*n* = 24). (**B**): Whole sample: *n* = 82; super taster, ST: *n* = 32; medium taster, MT: *n* = 31; non-taster, NT: *n* = 19. Genotypes of *CD36 rs1761667*: GG (*n* = 13), GA (*n* = 49) and AA (*n* = 19); genotypes of *CD36 rs1984112*: GG (*n* = 9), GA (*n* = 49) and AA (*n* = 23). (**C**): Whole sample: *n* = 85; super taster, ST: *n* = 33; medium taster, MT: *n* = 33; non-taster, NT: *n* = 19. Genotypes of *CD36 rs1761667*: GG (*n* = 14), GA (*n* = 51) and AA (*n* = 19); genotypes of *CD36 rs1984112*: GG (*n* = 9), GA (*n* = 52) and AA (*n* = 23).

The relationships between BMI and fatty acid detection thresholds are shown in Figure 2. For oleic acid (Figure 2A), BMI was positively correlated with detection threshold in the whole sample (r = 0.279, *p* = 0.009) and in NTs (r = 0.585, *p* = 0.0085). No significant correlations were found in other taster groups or genotype categories (*p* > 0.05). For linoleic acid (Figure 2B), a significant association with BMI was found only in NT participants (r = 0.574, *p* = 0.010), while no associations were observed in other groups (*p* > 0.05). For palmitic acid (Figure 2C), BMI was positively correlated with detection threshold in the whole sample (r = 0.299, *p* = 0.005), as well as in MT (r = 0.384, *p* = 0.027) and NT participants (r = 0.742, *p* = 0.0003). Significant positive correlations were also observed in individuals carrying the AA genotype of *CD36 rs1761667* (r = 0.542, *p* = 0.0165) and *CD36 rs1984112* (r = 0.515, *p* = 0.012), while no associations were observed in other genotypes (*p* > 0.05).

The relationships between BMI and fatty acid detection thresholds, stratified by sex, are shown in Figure S2. No significant association was observed between BMI and oleic acid detection threshold in females (*p* > 0.05; Figure S2A), whereas a positive correlation was found in males (r = 0.391, *p* = 0.048; Figure S2A,B). Linoleic acid detection threshold was not associated with BMI in females and males (Figure S2C,D). In addition, palmitic acid detection threshold was positively correlated with BMI in females (r = 0.298, *p* = 0.023) and males (r = 0.476, *p* = 0.012) (Figure S2E,F).

No significant associations were observed between BMI and caloric intake (Figure S3A), carbohydrate intake (Figure S3B), or fat intake (Figure S3C) (*p* > 0.05).

### 3.2. Multiple Regression Analyses

To identify independent predictors of BMI, stepwise multiple regression analyses were performed, including taste-sensitivity measures, sweet- and lipidic-genetic variables, and taster status as independent variables (Table 1). Accordingly, total taste strips, palmitic acid threshold, *TAS1R3 rs307355*, and oleic acid threshold were included in the model. The overall model predicted 18.83% of the variance in BMI. Total taste strips and palmitic acid threshold were significant contributors to the model, predicting 15.02% of the variance in BMI. Total taste strips showed an inverse association with BMI, whereas palmitic acid threshold displayed a positive association. *TAS1R3 rs307355* and oleic acid threshold also contributed independently to BMI variability.

**Table 1:** Stepwise forward multiple regression models for BMI.

Variable	β (*p*)	Each Step R^2^ (*p*)	Interpretation
Total taste strips	−0.32 (0.031)	0.072 (0.031)	Higher number associated with lower BMI
Total taste strips	−0.33 (0.008)	0.150 (0.007)	Lower threshold associated with lower BMI
Palmitic acid threshold	+0.29 (0.022)		
Total taste strips	−0.32 (0.012)	0.169 (0.011)	Independent genetic influence
Palmitic acid threshold	+0.25 (0.049)		
*TAS1R3 rs307355*	+0.14 (0.250)		
Total taste strips	−0.32 (0.011)	0.183 (0.014)	Independent lipid-related effect
Palmitic acid threshold	+0.23 (0.074)		
*TAS1R3 rs307355*	+0.15 (0.213)		
Oleic acid threshold	+0.14 (0.239)		

Taste sensitivity measure, taster status, and sweet and lipidic genetic variants were used as predictors. Only the variables that are entered in the statistical model are shown. Overall model: R^2^ = 0.188; adj R^2^ = 0.133; *p* = 0.014.

To assess whether caloric intake mediates the relationship between taste perception and BMI, total caloric intake was included in the model as an independent variable (Table 2). Accordingly, total taste strips, palmitic acid threshold, taster status, oleic acid threshold, *TAS1R2 rs35374116*, and *TAS1R3 rs307355* were entered in the model. The overall model predicted 44.76% of the variance in BMI. Total taste strips, palmitic acid threshold, and taster status were the significant contributors in the model, predicting 38.02% of the variance in BMI. Total taste strips showed a strong inverse association with BMI, palmitic acid threshold showed a direct association, and taster status showed an independent group-related effect on BMI, explaining an additional 7.2% of BMI variance beyond the other predictors. Oleic acid threshold, *TAS1R2 rs35374116*, and *TAS1R3 rs307355* also contributed independently to BMI variability.

## 4. Discussion

The present study demonstrates that taste sensitivity, assessed across global, sweet, and lipid modalities, is significantly associated with BMI, with a consistent inverse relationship across all sensory domains, mostly in specific taster and genotype groups. The inverse association between taste sensitivity and BMI is consistent with previous evidence showing that individuals with heightened sensory perception tend to exhibit lower body weight and healthier eating behaviors. Taste perception plays a fundamental role in dietary regulation, as it provides information about the nutrient composition and energy content of foods, influencing both food choice and intake behavior regulation [3,4,5]. Enhanced taste sensitivity may increase the perceived intensity of food stimuli and promote earlier satiety, thereby reducing overall intake [56]. In addition, genetic variability in taste receptors has been shown to contribute to interindividual differences in taste perception, dietary behavior, and BMI [3,7,14].

Specifically, results of correlation analyses revealed a robust inverse association between total taste sensitivity and BMI, particularly among STs, as well as in the overall sample. Similarly, sweet taste sensitivity showed consistent negative associations with BMI across both phenotypic and genotypic stratifications, mostly in ST participants and those carrying the sensitive genotype of *TAS1R2 rs35374116*, *TAS1R2 rs12033832*, *TAS1R3 rs307355*, and *GNAT3 rs7792845* SNPs. The observed inverse association between sweet taste sensitivity and BMI, particularly across *TAS1R* genotypes, seems to support the role of sweet taste receptors in energy regulation. The T1R2/T1R3 receptor complex is responsible for sweet taste perception and has been linked to sugar intake, food preference, and obesity risk [14,16]. Variants in *TAS1R* genes influence sweet perception and may modulate dietary behavior and long-term energy balance. Importantly, sweet taste receptors are also expressed in extra-oral tissues, including the gastrointestinal tract, where they regulate glucose sensing, hormone secretion, and metabolic responses [24].

Correlation analyses also showed a higher sensitivity to fatty acids, particularly palmitic acid, associated with lower BMI, both in the overall sample and in specific subgroups: NT participants and those carrying the insensitive genotype of the two SNPs of the *CD36* gene. These results are consistent with evidence supporting the existence of a fat taste modality mediated by receptors such as CD36 [29]. CD36 is involved in oral lipid sensing and has been associated with fat sensitivity and preference, dietary fat intake, and obesity-related traits [32,33,34,35,36,57]. Previous studies have shown that individuals with reduced fat taste sensitivity tend to consume more fat-rich foods and exhibit higher BMI [34]. Therefore, increased lipid sensitivity might serve as a protective factor, enhancing the detection of energy-dense foods and limiting overconsumption. Moreover, individuals with altered CD36 function or higher lipid detection thresholds have been shown to exhibit greater preference for fatty foods and higher BMI, supporting a mechanistic link between fat perception and body weight regulation. However, an additive genetic model does not confirm these associations by showing that carriers of the insensitive allele of *CD36 rs1761667* have higher BMI, hip circumference, and weight compared to carriers of the sensitive allele [58]. The sex-segregated correlation analyses indicate that the associations between BMI and total/sweet taste sensitivity were observed specifically in females, while those with lipid taste were observed mostly in males.

Taken together, these findings suggest that high sugar and lipid sensitivity might act as a protective mechanism against excessive energy intake and weight gain. Interestingly, the association between taste sensitivity and BMI showed a modality-specific pattern. For sweet taste, the relationship was strongest among STs and carriers of sensitivity-related *TAS1R* genotypes, suggesting that a highly responsive sensory system could enhance the behavioral relevance of sweetness perception. In contrast, for lipid perception, associations were more evident in NTs and individuals with less sensitive *CD36* variants, possibly indicating that reduced fat sensitivity could amplify variability in lipid-driven dietary choices and metabolic responses, possibly amplifying its observable relationship with BMI. It is interesting to note that the total caloric intake, as well as carbohydrate and fat intake, did not correlate with BMI.

The multiple regression analysis confirms and extends these results, showing a stable and interpretable model in which both overall taste and lipid sensitivity were inversely associated with BMI. Additionally, *TAS1R3 rs307355* independently contributed to BMI variability, suggesting a role of genetic taste components beyond taste perception measures. Importantly, total caloric intake did not mediate these associations. In fact, when caloric intake was included in the regression model, it did not remain as an independent predictor of BMI, while taste sensitivity variables and genetic factors retained their significance without attenuation of the coefficients, suggesting that the relationship between taste perception and BMI is not primarily mediated by total caloric intake in this context. Additionally, in this model, taster status significantly contributes to BMI variability, suggesting an independent taster group-related effect. *TAS1R2 rs35374116* showed a protective effect, and *TAS1R3 rs307355* was also included in the model, confirming its role in BMI variation. We acknowledge that hierarchical regression represents an alternative theory-driven approach. However, given the exploratory nature of identifying independent sensory and genetic predictors of BMI, we considered forward stepwise regression the most appropriate method for the objectives of the present study.

These findings suggest that qualitative aspects of diet, sensory perception, and metabolic factors play a key role beyond total energy intake. A plausible explanation for these direct effects is the presence of taste receptors in extra-oral tissues. Taste receptors are expressed in several organs, including the gastrointestinal tract, pancreas, and adipose tissue, where they contribute to nutrient sensing, hormone secretion, and metabolic regulation [24,58]. These extra-oral taste receptors could be part of an integrated physiological network influencing BMI independently of conscious dietary intake. In this context, taste should be considered a biological regulator of energy balance and metabolism, not only a determinant of food preference.

## 5. Conclusions

The present study provides novel evidence supporting a central role of taste perception in the regulation of body weight. In particular, the findings indicate that greater taste sensitivity is consistently associated with lower BMI, suggesting a protective role of enhanced sensory function. Importantly, this relationship was found to be independent of total caloric intake, suggesting that the influence of taste on BMI extends beyond simple differences in the amount of energy consumed. In this context, our results suggest that taste perception could contribute to body weight regulation through a more complex interplay of mechanisms, potentially involving food selection, dietary composition, and metabolic responses. In addition, the contribution of genetic polymorphisms in taste receptors, such as *TAS1R* and *CD36*, reinforces the biological basis of these associations. These genetic factors may influence not only sensory perception, but also physiological processes related to nutrient sensing and metabolism. This supports the emerging view of the oral and extra-oral gustatory system as part of a broader network regulating energy balance at multiple levels.

From a clinical and nutritional perspective, these findings underscore the limitations of models that focus exclusively on caloric intake to explain BMI. Instead, they suggest that individual differences in sensory perception and genetic background should be considered as key determinants of eating behavior and metabolic health. These implications are particularly relevant to the development of personalized nutrition strategies, where taste-sensitivity profiling and genetic information could help identify individuals at higher risk of obesity and guide targeted interventions.

Future research should further investigate the longitudinal relationships between taste sensitivity and weight change, as well as the underlying mechanisms linking sensory perception to metabolic outcomes. Although participants abstained from food and beverages for at least 2 h before taste testing, we cannot exclude the possibility that recent dietary intake influenced taste sensitivity measurements. Future studies may benefit from longer fasting periods or standardized pre-test meals to further control for acute dietary effects.

## Figures and Tables

**Table 2:** Stepwise forward multiple regression models for BMI, including caloric intake as an independent variable.

Variable	β (*p*)	Each Step R^2^ (*p*)	Interpretation
Total taste strips	−0.37 (0.016)	0.135 (0.016)	Strong inverse association with BMI
Total taste strips	−0.42 (0.003)	0.308 (<0.00)	Direct positive association with BMI
Palmitic acid threshold	+0.42 (0.003)		
Total taste strips	−0.40 (0.003)	0.380 (<0.00)	Group-related effect on BMI
Palmitic acid threshold	+0.41 (0.003)		
Taster status	+0.27 (0.042)		
Total taste strips	−0.39 (0.005)	0.404 (<0.00)	Independent lipid-related effect
Palmitic acid threshold	+0.41 (0.003)		
Taster status	+0.29 (0.030)		
Oleic acid threshold	+0.16 (0.226)		
Total taste strips	−0.39 (0.005)	0.429 (<0.00)	Protective genetic effect
Palmitic acid threshold	+0.35 (0.016)		
Taster status	+0.26 (0.048)		
Oleic acid threshold	+0.18 (0.163)		
*TAS1R2 rs35374116*	−0.17 (0.226)		
Total taste strips	−0.38 (0.005)	0.448 (0.001)	Independent genetic influence
Palmitic acid threshold	+0.29 (0.048)		
Taster status	+0.27 (0.043)		
Oleic acid threshold	+0.20 (0.137)		
*TAS1R2 rs35374116*	−0.17 (0.223)		
*TAS1R3 rs307355*	+0.15 (0.281)		

Taste sensitivity measure, taster status, and sweet and lipidic genetic variants and caloric intake were used as predictors. Only the variables that are entered in the statistical model are shown. Overall model: R^2^ = 0.4476; adj R^2^ = 0.3529; *p* = 0.0013.

## Data Availability

The data presented in this study are available on request from the corresponding author. Data are not publicly available due to ethical restrictions.

## Supplementary Materials

The following supporting information can be downloaded at https://www.mdpi.com/article/10.3390/nu18152459/s1. Figure S1. Scatterplots showing the Pearson linear correlation between total taste strips and BMI in females (A) and males (B). Scatterplots showing the Pearson linear correlation between the sweet taste strips and BMI in females (C) and males (D). Pink area indicates a statistical difference (r > −0.367, *p* < 0.008). Figure S2. Scatterplots showing the Pearson linear correlation between BMI and the oleic acid threshold in females (A) and males (B), linoleic acid threshold in females (C) and males (D), and palmitic acid threshold in females (E) and males (F). Green area indicates a statistical difference (r > 0.391, *p* < 0.048). Figure S3. Scatterplots showing the Pearson linear correlation between BMI and caloric intake (A), carbohydrate intake (B) and fat intake (C).
