# Supplementary material for: Taste Sensitivity Is Inversely Associated with Body Mass Index (BMI) Independently of Caloric Intake: Evidence from Sensory and Genetic Analyses
Source: Nutrients. 2026 Jul 27;18(15):2459. doi: 10.3390/nu18152459 (PMC13467708; doi:10.3390/nu18152459)
Supplement: Supplementary file 1 [file nutrients-18-02459-s001.zip › nutrients-4428732-supplementary.pdf]

SUPPLEMENTARY MATERIALS

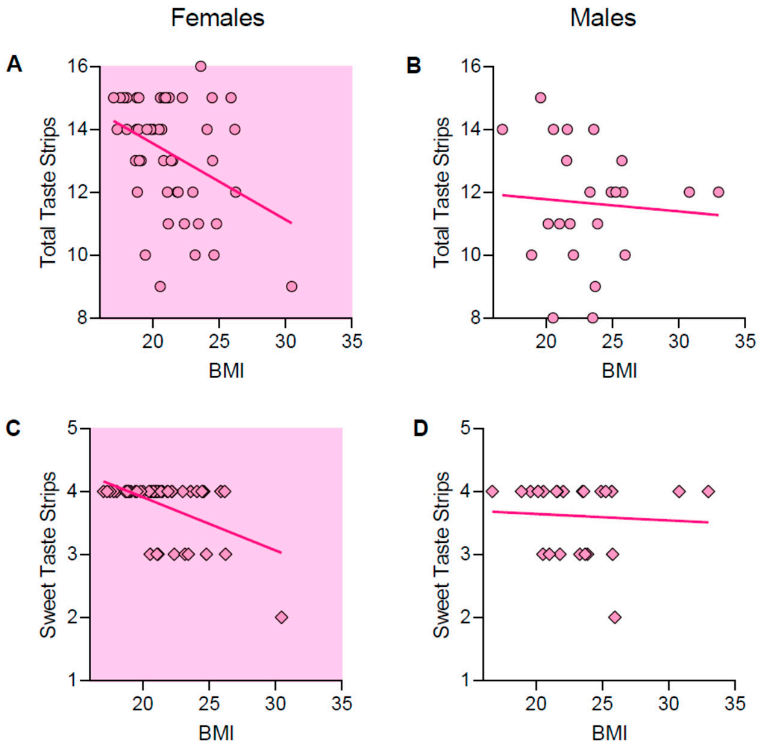

Figure S1. Scatterplots showing the Pearson linear correlation between total taste strips and BMI in females (A) and males (B). Scatterplots showing the Pearson linear correlation between the sweet taste strips and BMI in females (C) and males (D). Pink area indicates a statistical difference (r > - 0.367, *p* < 0.008).

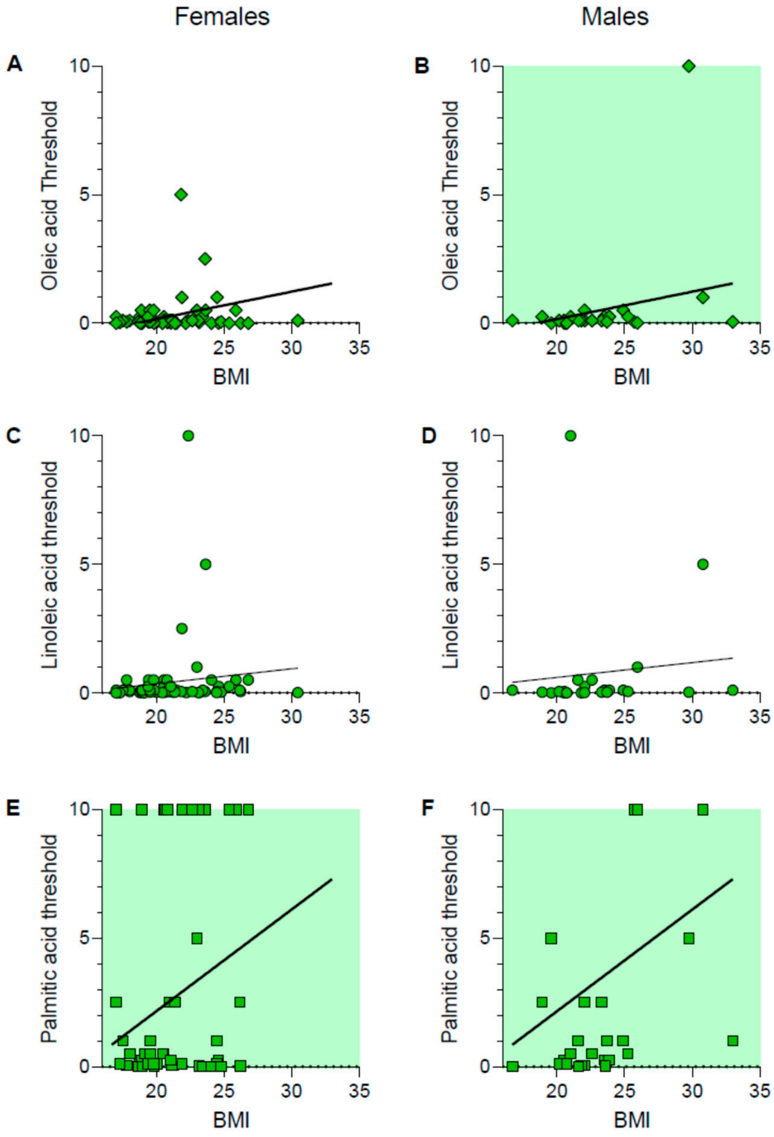

Figure S2. Scatterplots showing the Pearson linear correlation between BMI and the oleic acid threshold in females (A) and males (B), linoleic acid threshold in females (C) and males (D), and palmitic acid threshold in females (E) and males (F). Green area indicates a statistical difference (r > 0.391, p < 0.048).

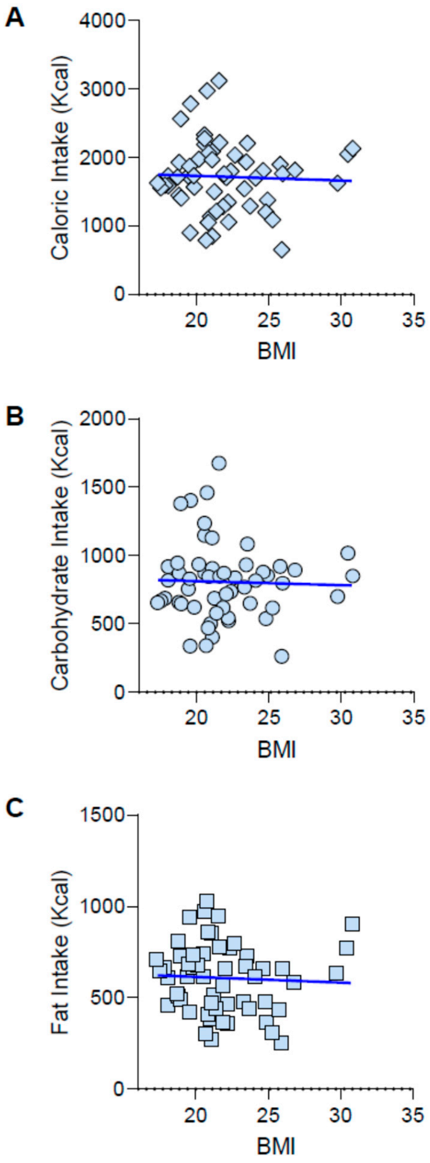

Figure S3. Scatterplots showing the Pearson linear correlation between BMI and caloric intake (A), carbohydrate intake (B) and fat intake (C).
